# Variable Cognition in ABM Decision-Making: An Application to Livestock Vaccine Choice

**DOI:** 10.3389/fvets.2020.564290

**Published:** 2020-10-15

**Authors:** Richard A. Iles, Matthew J. Sottile, Ofer Amram, Eric Lofgren, Craig S. McConnel

**Affiliations:** ^1^School of Economic Sciences, Washington State University, Pullman, WA, United States; ^2^Paul G. Allen School for Global Animal Health, Washington State University, Pullman, WA, United States; ^3^Department of Mathematics, Washington State University, Vancouver, WA, United States; ^4^Department of Nutrition and Exercise Physiology, Washington State University, Spokane, WA, United States; ^5^Department of Veterinary Clinical Sciences, Washington State University, Pullman, WA, United States

**Keywords:** cognition, pastoralist, decision-making, vaccine, agent-base model, Kenya

## Abstract

Modeling realistic human decision-making is an important feature of good policy design processes. The use of an agent-based modeling framework allows for quantitative human decision-models that assume fully rational agents. This research introduces a dynamic human decision-making sub-model. The parameterisation of human memory and “rationality” in a decision-making model represents an important extension of decision-making in ABMs. A data driven model of herd movement within a dynamic natural environment is the context for evaluating the cognitive decision-making model. The natural and human environments are linked via memory and rationality that affect herdsmen decision-making to vaccinate cattle using a once-for-life vaccine (Rift Valley fever) and an annual booster vaccine (Contagious Bovine Pleuropneumonia). The simulation model uses environmental data from Samburu county, Kenya from 2004 to 2015. The cognitive parameters of memory and “rationality” are shown to successfully differentiate between vaccination decisions that are characterized by annual and once-for-life choices. The preliminary specifications and findings from the dynamic cognition–pastoralist agent-based model (*PastoralScape*) indicate that the model offers much to livestock vaccination modeling among small-scale herders.

## Introduction

The economic sustainability of traditional pastoralist modes of livestock management is threatened by environmental, political and cultural forces across East Africa (1, 2). The increased frequency of droughts in East Africa over the past 20-years has sorely tested the resilience of livestock dependent communities in the region (3, 4). The need to model the complex interaction between natural and human system, as they affect livestock, is a research topic deserving further attention (5). The role of human decision-making as it affects livestock health adds to the complexity of such systems.

The advent of behavioral economics, and the acknowledgment within economics that human decision-making is more heterogeneous than previously assumed, leads one to question the oft assumed “rational agent” hypothesis. A review of decision-making paradigms used in animal health demonstrates the wide use of qualitative and quantitative decision frameworks (6). Within a quantitative framework, Prospect Theory is a common means of identifying heterogeneous methods of decision-making (7, 8). The use of short-term measures of human cognition is another (Choi and Iles, under review). Empirical investigation of the role of short-term cognitive capacity on human decision-making is based on the Mullainathan and Shafir's (9) “scarcity thesis”. This thesis argues that financial stress affects human decision-making via changes in short-term cognition [(9, 10); (Iles et al., under review)].

Modeling realistic human decision-making is no less important with respect to animal health related decisions (6). However, the allure and utility of assuming fully rational agents remains strong. Constrained maximization/minimization approaches are limited in their ability to approximate the heterogeneity in human decision-making, necessary for good public policy modeling (11, 12). Whether simulation, Randomized Control Trials, or hypothetical scenarios are used to generate data, the fully rational agent is frequently assumed (13–16). This assumption of optimal decision-making is often demonstrated to be often unrealistic in the case of livestock management among the global poor (17).

Agent or individual-based modeling (ABM) provides a tractable means of analyzing the effects of interconnected dynamics of human and natural environments on household decision-making and resource allocation. Such a modeling framework allows for quantitative human decision-models that do not assume fully rational agents. Existing ABMs concerned with the dynamic environments of pastoralists in East Africa are typically concerned with tribal conflict (18, 19), decision-making (20), humanitarian crises (21), risk-sharing and cooperation (22–24), and climate change adaptation (25). While the present research also uses an ABM framework, its primary contribution is the introduction of a dynamic human decision-making sub-model. The parameterization of human memory and “rationality” in a decision-making model represents an important extension of decision-making in ABMs. The preliminary *PastoralScape* model presented here also provides insight into possible policy relevant extensions.

The current *PastoralScape* ABM documents a data driven model of herd movement within a dynamic natural environment. The natural and human environments are linked via memory and rationality that affect herdsmen decision-making to vaccinate cattle for Rift Valley Fever (RVF) and Contagious Bovine Pleuropneumonia (CBPP). The simulation model uses environmental data from Samburu county, Kenya, from 2004 to 2015. The difference in the frequency of vaccinations for each disease provides a means for assessing the effects of memory and “rationality” on one-time and repeated decision-making. Toward this end, the ABM introduces a Random Field Ising Model (RFIM) to estimate the binary choice to vaccinate. Such a decision is modeled in the context of the uncertainty of disease transmission dynamics of each disease.

## Materials and Methods

This section is organized using the Overview, Design Concepts and Details (ODD) structure for reporting ABMs (26). This reporting structure aims to provide a consistent format in reporting the objectives, structure and data used. In light of the need to more adequately capture the details of human decision-making, a revision to this protocol was proposed and referred to as ODD+D (27). The addition of the “+D” represents the addition of human decision making within the ABM. As a means of reporting the use of a RFIM in our model, where feasible, we follow the OOD+D protocol.

### Overview

#### Purpose

The current preliminary *PastoralScape* simulation model aims to assess the medium-run dynamics of livestock vaccine decisions for two livestock diseases (RVF and CBPP). Pastoralist heads-of-households in Samburu county, Kenya, are assumed to have varying levels of cognitive ability. The medium-run is defined as an eleven-year period. For the purposes of clearly communicating the innovative human decision making sub-model, along with its interaction with the other sub-models, the livelihoods of human agents are simplified. All heads-of-households solely practice pastoralist cattle raising, cattle are not sold or traded, and there is no income within the model. Heads-of-households only make decisions related to livestock vaccination. Herdsmen only makes decisions about the movement of herds. Future development of the *PastoralScape* model will include a local economy and more diversified streams of household income.

The preliminary model assumes that all heads-of-households are homogenous. As a result, their utility functions, that drive their vaccine decision making, are fixed across agents. This assumption is made in order to clearly communicate the decision making sub-model and document the basic interactions between sub-models. While the *PastoralScape* model will ultimately enable the modeling of heterogeneous agents, the current version of the model is not designed to do so. Nevertheless, the innovativeness of the human decision making sub-model in the ABM readily allows for heterogeneity.

The use of RVF and CBPP as examples for modeling human vaccination decision-making provides two separate decisions that involve contrasting frequency and assumed levels of disease risk. Uncertainty is an important feature that drives decision-making (7, 8). The RVF vaccine's efficacy is once-for-life, administered to protect against sporadic outbreaks reported across Kenya. CBPP requires an annual booster with efficacy of approximately 6-months. The differing patterns of decision-making for these vaccines are assumed to influence vaccination outcomes. Outbreaks of RVF are closely linked to precipitation and mosquito populations (28–30). As a result, expectation of RVF outbreaks may follow a medium and long-run cyclical pattern. On the other hand, outbreaks of CBPP are less clearly predicted. Therefore, the assumed levels of uncertainty associated with disease outbreaks also differ between the two livestock diseases. No recent outbreaks of RVF in Samburu county have been recorded. The county maintains surveillance of CBPP despite the absence of a recent outbreak (31).

#### Entities, State Variables, and Scales

Three types of agents are modeled. These are: (a) heads-of-household, (b) cattle, and (c) herdsman. It is assumed that all heads-of-households engage in small-scale pastoralist cattle raising. There are no sedentary crop farmers or cattle rangers in the model. In keeping with the cultural practices of pastoralists in Samburu county, Kenya, each head-of-household has a herdsman to manage cattle. Culturally, these herdsmen are young men. Each agent type is defined by a set of attributes. Heads-of-households are defined according to the parameters of the RFIM and only make decisions as to whether to vaccinate livestock. These parameters include: memory (μ), degree of rationality (β), latent willingness to make a vaccination decision (f_i_), access to public information [F(t)], and a social network (see Design Concept section for further details of the RFIM). Cattle are defined by: (i) sex, (ii) age, (iii) health, (iv) SIRV disease state, and (v) location. Cattle are modeled as individual agents, not as a single herd. Herdsmen are defined by (i) their co-location with livestock and their movement, and (ii) their knowledge of foraging conditions within a limited radius. Cattle and herdsmen are able to move spatially, while heads-of-households are fixed and uniformly distributed between five village locations.

The time-step used to progress the changes in the simulation environment, movement of livestock, and human decisions is 7 days. The time-step of the SIRV sub-model is scaled from daily. Environmental data (including Normalized Difference Vegetation Index (NDVI), Foraging Condition Index and precipitation measurement) are assumed constant during each month.

### Design Concepts

#### Basic Principles

The coupling of natural and human environments in this ABM provides an important set of relationships that drive assumed changes in financial and mental stress of pastoralist households. The parameterisation of head-of-household memory and “rationality” provides flexibility to model two important aspects of cognitive ability (32–34). Human cognitive ability or capacity are believed to change over time due to stress, anxiety and the perception of these (10, 35, 36). According to Mullainathan and Shafir's “scarcity thesis,” perceptions of household financial stress act as a tax on cognitive capacity [(9); (Iles et al., under review)]. Therefore, the parameterisation of two aspects of human cognition allows for more realistic modeling of the cognitive dynamics in discrete decision-making. Although the calibration of empirical data capturing short-term changes in cognitive capacity (i.e., fluid intelligence and working memory capacity) is not included in this preliminary *PastoralScape* model (see 38 for details of empirical data following the 2016–2017 drought in Samburu), future work will do so. The flexibility of the proposed human decision making sub-model motivates its introduction to ABMs in this paper.

**Figure 1:**
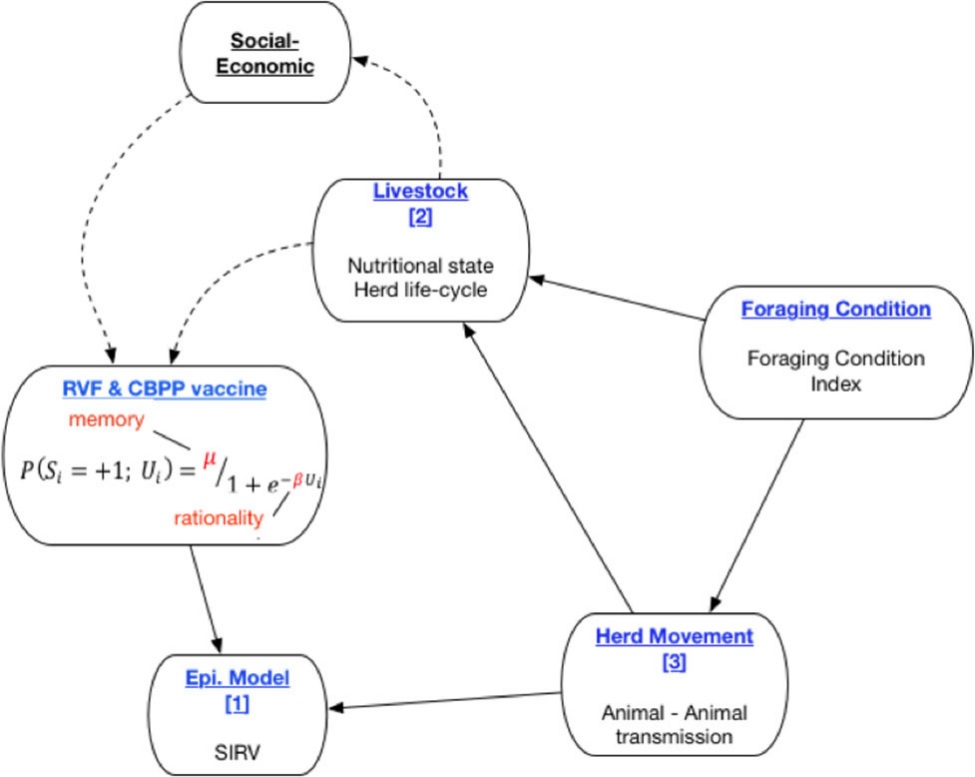
Overview of preliminary PastoralScape sub-models.

The *PastoralScape* model is depicted in Figure 1. The sub-components of the current model are titled in blue. The solid connecting lines reflect the direction of interaction between model sub-components captured in *PastoralScape*. Three sub-models are numbered (1, 3). Foraging Condition is calculated independently of *PastoralScape*. The decision to vaccine livestock against RVF and CBPP is determined by the cognitive parameters μ (memory) and β (rationality). The dotted lines depict proposed extensions to the *PastoralScape* model connecting livestock and household socio-economic variables to dynamic changes to μ and β (Choi and Iles, under review; Iles et al., under review). The rest of the paper focuses on sub-components and the solid line relationships. A more detailed explanation of the interaction between sub-models is given when explaining the respective sub-models.

#### Adaptation and Learning

Head-of-household decision-making is modeled as a nested process and one that follows an existing specification (37). Decision-making is based on an Ising model, which incorporates individual willingness to act (*f*_*i*_), public information (*F*), and network pressure (*J*_*ij*_), to determine decision to sell (S: +1, −1) cattle.

(1) $$\begin{array}{l} U_{i}\left(t\right)=\;f_{i}+F\left(t\right)+\sum_{j\epsilon \Upsilon_{i}}J_{ij}S_{j}\left(t-1\right), \end{array}$$

where *U*_*i*_ is perceived incentive to act, Υ_*i*_ is the neighborhood of agent *i*, and *t* is time (37). Agents act (+1, −1) when *U*_*i*_ is greater than some unobserved threshold. Ising models are frequently used in economics to model the effect of network pressure on decision-making (38–40). In this paper, we assume for simplicity that U_*i*_ is identical for every head-of-household and fixed through time. Individual willingness to act (f_*i*_), public information [F(*t*)], and network pressure (J_*ij*_) play no role in influencing the results of this paper. The values of these parameters are fixed across all runs and do not affect results.

By incorporating the Ising specification from equation 1 into a logit structure (see equation 2), the parameters of μ and β are created to *tune* resulting probabilities of a binary choice. The parameter μ ranges between zero and one and captures the degree of memory of the immediate past decision. For a head-of-household in the model, a degree of memory (μ) equal to one would imply that they remember exactly if and when they last administered an RVF or CBPP vaccine. A degree of memory equal to zero would imply that decision makers have no memory of the immediate past decision. In this scenario each decision is independent of the previous. The β parameter controls the amount of irrationality in the decision process (*Type* 2:β → ∞ deterministic, and *Type* 1:β → 0 random process). The degree of determinism used by the human agent is referred to as ‘rationality'. This language follows that used by Bouchaud (37).

(2) $$\begin{array}{l} P\left(S_{i}=+1;U_{i}\right)=\frac{\mu }{1+e^{-\beta U_{i}}}. \end{array}$$

In the current preliminary model, the cognition parameters are held constant across the population of heads-of-households. That is, in this paper, we do not model heterogeneity of cognition parameters among the different heads-of-households. We assume homogenous parameters across all heads-of-households. However, this may not always be the case. Heterogeneity in the setting of μ and β is possible in future applications of the model. As individuals' perception of their current and future financial status differ by income and livestock loss (in the case of pastoralists), heterogeneous and dynamic cognition parameters could be incorporated in the future (Choi and Iles, under review; Iles et al., under review).

### Details

#### Input Data

**Figure 2:**
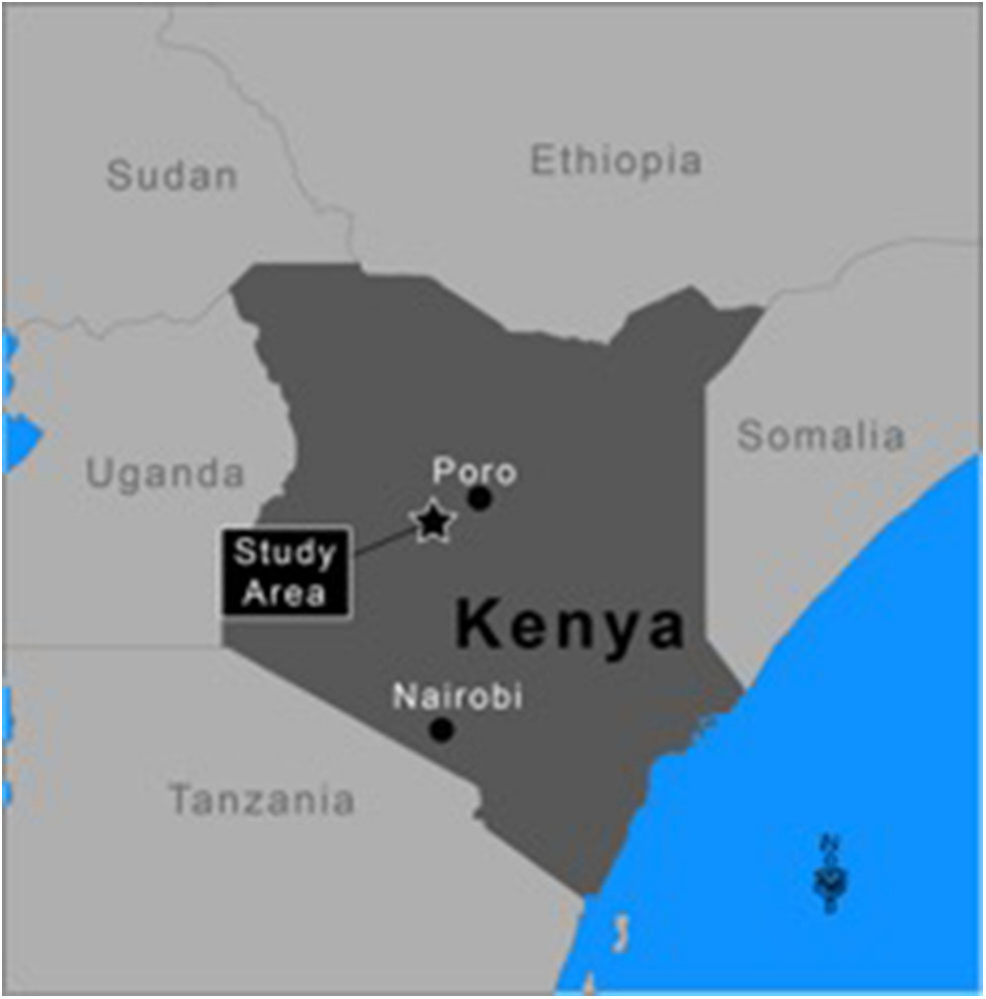
Simulated world reflecting the natural environment of Samburu county.

**Figure 3:**
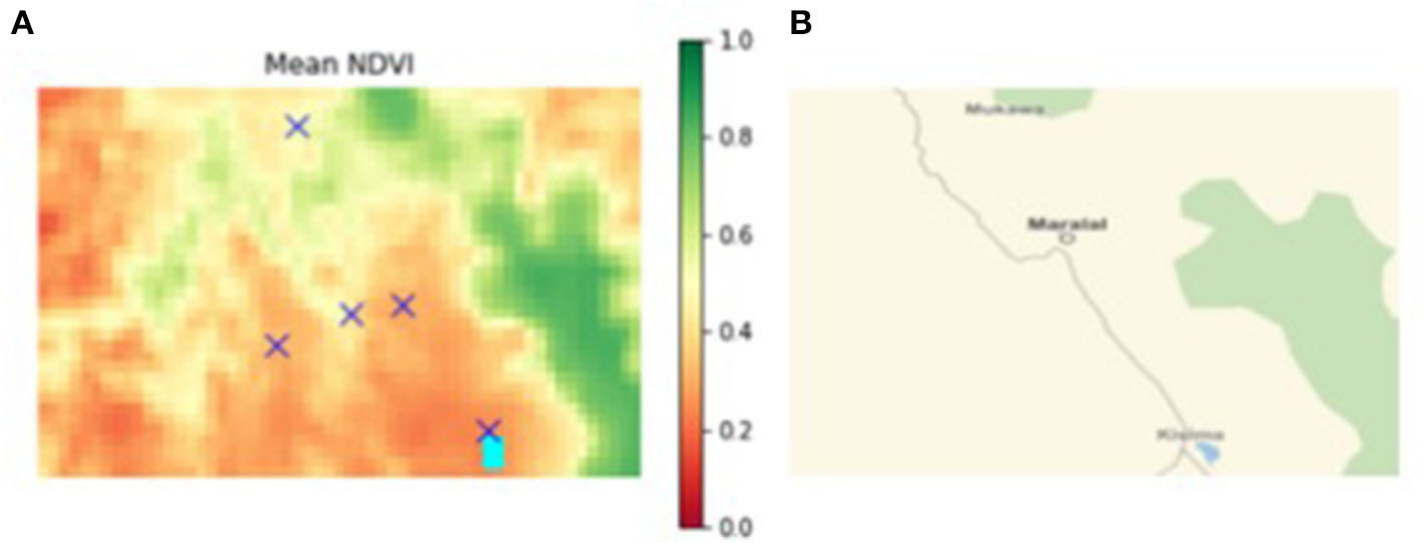
**(A)** Mean NDVI of south-western Samburu county (location of sample villages denoted by “X”), **(B)** major road network connecting sample villages Kisima (south) to Poro (north), through Maralal.

The simulated “world” uses environmental data from south-western Samburu county, Kenya, from 2004 to 2015 (Figure 2). The “world” is constructed as a rectangular grid (35 × 55 km), which comprises 1 by 1 km sized cells. Village locations are fixed and align with the actual locations of surveyed villages (Figure 3). Agents reside either permanently (villages and heads-of-households) or temporarily (herdsmen and herds) within a given cell. When located within a cell agents have access to all resources co-located in the cell (i.e., other human agents and forage). It is assumed that when more than one herd and herdsman are co-located on a given cell they interact. Overlaying this rectangular “world” is a fixed social network of relationships between heads-of-households. All heads-of-households are linked to each other. For the purposes of this preliminary *PastoralScape* model, the importance of relationship weighting is equal across the social network. Due to this assumed social network weighting, the extent of the social network (either global or village based) has marginal effect on vaccination decisions.

The time period (2004–2015) coincided with three distinct droughts. The 2010–2011 and 2015–2016 droughts affected much of East Africa (3). A more localized drought in 2005–2006 affected Kenya (41). Figure 2 locates the 35 km by 55 km area from which NDVI, FCI and precipitation data are drawn. This area of Samburu county is classified as semi-arid. The Samburu pastoralists are traditionally semi-nomadic, moving their cattle in dry seasons or drought to find better forage (42). The NDVI and precipitation data is from Google Developers (43, 44). Aggregate FCI data is used from the PLEWS model (45). The FCI data is scaled by the 1 km by 1 km grid NDVI values. FCI is used to reflect available livestock forage as it is believed to provide a more accurate measure than NDVI.

Household survey data from residents of five villages depicted in Figure 3 inform the selection of average herd size per household. This survey also contains repeated measures of short-term cognition. Three rounds of data were collected from each village between October 2017 and September 2018 (46). This period coincided with the end of the 2015–2016 drought that gripped much of East Africa.

#### Submodels

**Figure 4:**
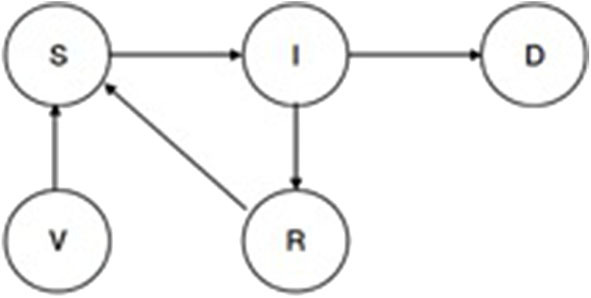
SIRV epidemiological transmission model.

A disease transmission sub-model [see sub-model (1) in Figure 1] uses a basic Susceptible, Infected, Recovered, Vaccinated (SIRV) structure. RVF and CBPP each have a separate SIRV sub-model. The transmission probabilities estimated in two papers are used to inform the selection of sub-model parameters (47, 48). Figure 4 outlines the structure of the disease sub-model, while Table 1 details the transition probabilities used for each disease. The use of Markov disease transmission models is common in ABMs (21). The V to S transition corresponding to a vaccine wearing off is modeled based on the time-since-vaccination transition probability dependent on the time spent in the vaccinated state. A herds' disease susceptibility is dependent on a collocated animal having the disease. The mixing of cattle affects herds' susceptibility (transition from S to I). All cattle within a village are located on the same cell. Once herds move away from their home village their susceptibility is dependent on collocating on a cell with other herds. Therefore, herds' location, relative to other herds, within the grid space affect the likelihood of disease transmission. This results in the S to I transition to turn on and off, subject to the collocation of herds. Livestock susceptibility to RVF and CBPP does not account for the age or health profile of animals.

**Table 1:** Markov transition probabilities.

	**RVF**	**CBPP**
Prob_si	0.14	0.024
Prob_ir	0.0001	0.0045
Prob_id	0.3	0.009
Prob_rs	0.0	0.0
Prob_vs	0.0	0.00091

*s, susceptible; i, infected; r, recovered; d, death; v, vaccinated*.

In addition to the risk of dying from RVF or CBPP, cattle may also die of old age or starvation. The non-disease related health of cattle is a separate sub-model [see sub-model (2) in Figure 1]. Non-disease health is measured along a zero to one continuum with zero representing death and one perfect health. Livestock require 0.1 units of feed per day. Available forage is calculated as the ratio of current available FCI relative to the historical average for the same place. When the ratio is one or greater, cattle are guaranteed to have food requirements met. For values less than one, cattle receive less than their required food, and thus livestock health degrades by 0.0175 per week. Symmetrically, if cattle receive more than their required food, their health improves by 0.0175 per week. Changes in livestock nutritional in-take is assumed uniform across a single herd. The age, gender, and health of cattle effects fertility. The occurrence of droughts during the timespan of the model serves two purposes. First, they help to diversify the causes of livestock death in the model to provide a sharper contrast with disease related deaths. Second, future versions of *PastoralScape* will use the experience of droughts to act as a stress on cognitive capacity. The experience of such stress will provide a heterogeneous cognitive shock to heads-of-households.

Herd movement [see sub-model (3) in Figure 1] is determined by herdsmen who have a 20-km radius of knowledge about surrounding foraging condition and long-term water availability. Herdsmen decide to move their cattle to a neighboring cell when the foraging condition of their village cell falls below the long-term average. Herdsmen continue to move as long as neighboring cells have higher FCI measures. After which time they return to their home village. The immediate effect of herds moving away from their home village is improved nutritional health. However, depending on the severity of a drought, herd movement within the grid-space may not continue to protect a herds' health. Each head of household manages 10 herdsmen, and each herdsmen has a herd of 20 cattle. Thus, each head of household is assumed to own 200 cattle. We simulate ten heads-of-households, for a total of 2,000 head of cattle simulated. The assumption that herdsmen have knowledge of surrounding foraging conditions is also reflected in the independent HerderLand ABM developed by Kennedy et al. (49). In the *PastoralScape* model the transition probability of moving from Susceptible to Infected is effective only when herds are collocated on the same cell. It is assumed that disease mixes completely through a single herd if one of the animals contracts the disease.

## Results

**Figure 5:**
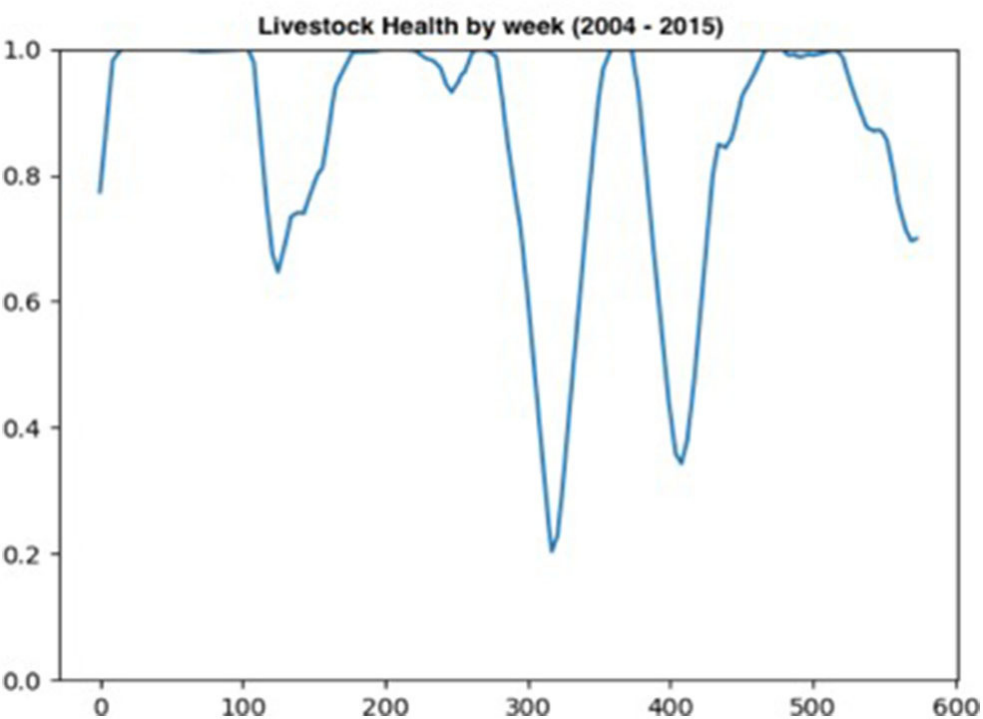
Changes in Livestock health per week over 11.5 years.

The livestock health sub-model captures the expected effects of the 2006, 2010–2011, and 2015 droughts on livestock health. Figure 5 plots the weekly aggregate measure of livestock health between 2004 and 2015. Two large drops in livestock health are observed in 2010 (approximately week 310) and 2012 (approximately week 410). Smaller declines in livestock health are recorded in 2006 (approximately week 110) and in 2015 (approximately week 0).

The effect of synthetic uniform changes (across separate simulation runs, not through time within a given run) in the assumed rationality (β parameter) of heads-of-households on livestock deaths due to RVF and CBPP are recorded in Table 2. Deaths due to the combined effects of RVF and CBPP diminish in absolute and relative terms as β (rationality) increases. As β increases from 0.0 (random) to 0.5 (more rational) the combined total of livestock disease deaths decreases from 744 to 581 out of ~2,430 head of cattle that die during the simulation. Results also remind us, though, that a trade-off exists between deaths by disease and old age. An increase in rationality reduces deaths due to disease, but also increases deaths due to old age.

**Table 2:** Causes of livestock death resulting under different assumed values for parameter beta (rationality).

		**Cause of Livestock Death**				
**beta**	**Old age**	**Starvation**	**RVF**	**Sum RVF + CBPP**	**CBPP**	**Total**
nill	1,428	9	635	1017	382	2,454
0.0	1,703	16	513	744	231	2,463
0.1	1,737	16	501	692	191	2,445
0.2	1,784	16	493	628	135	2,428
0.5	1,818	17	480	581	101	2,416
1.0	1,824	17	481	581	100	2,422
2.5	1,825	17	480	579	99	2,422
5.0	1,824	17	480	579	99	2,421

**Figure 6:**
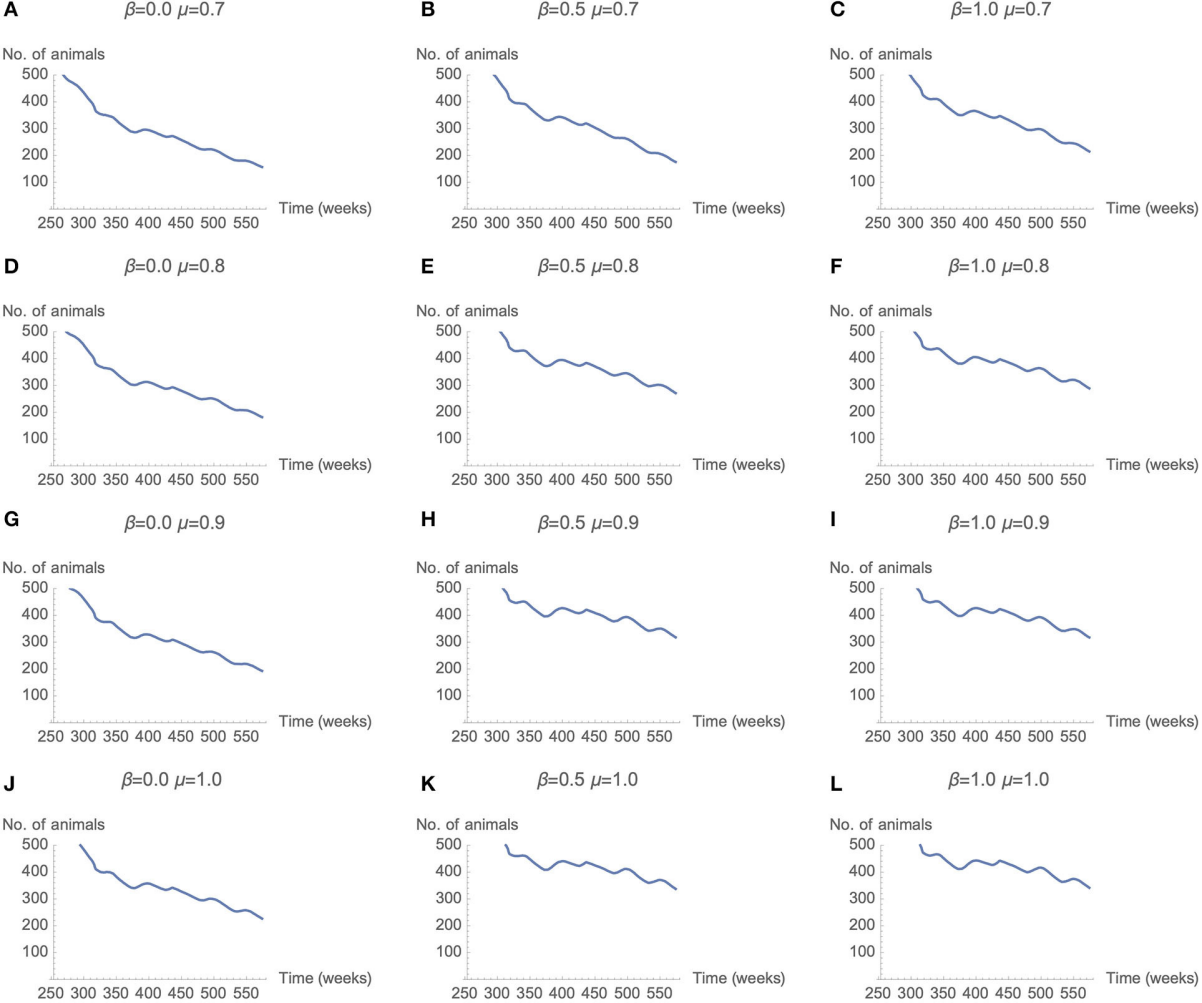
Total herd population over time as μ and β parameters change (50 seeds used per parameter combination).

Across a select range of μ (memory) and β (rationality) parameter combinations, a stable number of cattle is achieved. This stable level is achieved by altering the memory and rationality parameters, which results in changes to the number of livestock vaccinated for RVF and CBPP. Figure 6 presents the number of live cattle averaged over 50 simulation runs for each combination of μ and β. Each row of the matrix represents results for μ values 0.7, 0.8, 0.9, and 1. Each column represents results for β values 0, 0.5, and 1. The decline in the number of live cattle in the model declines over time. However, the rate of decline is more gradual as μ (memory) and β (rationality) parameter values increase. In Figures 6E,F,H,I,K,L the rate of decline appears most gradual. The parameter combinations of these sub-plots are β is ≥0.5 and μ is ≥0.8.

**Figure 7:**
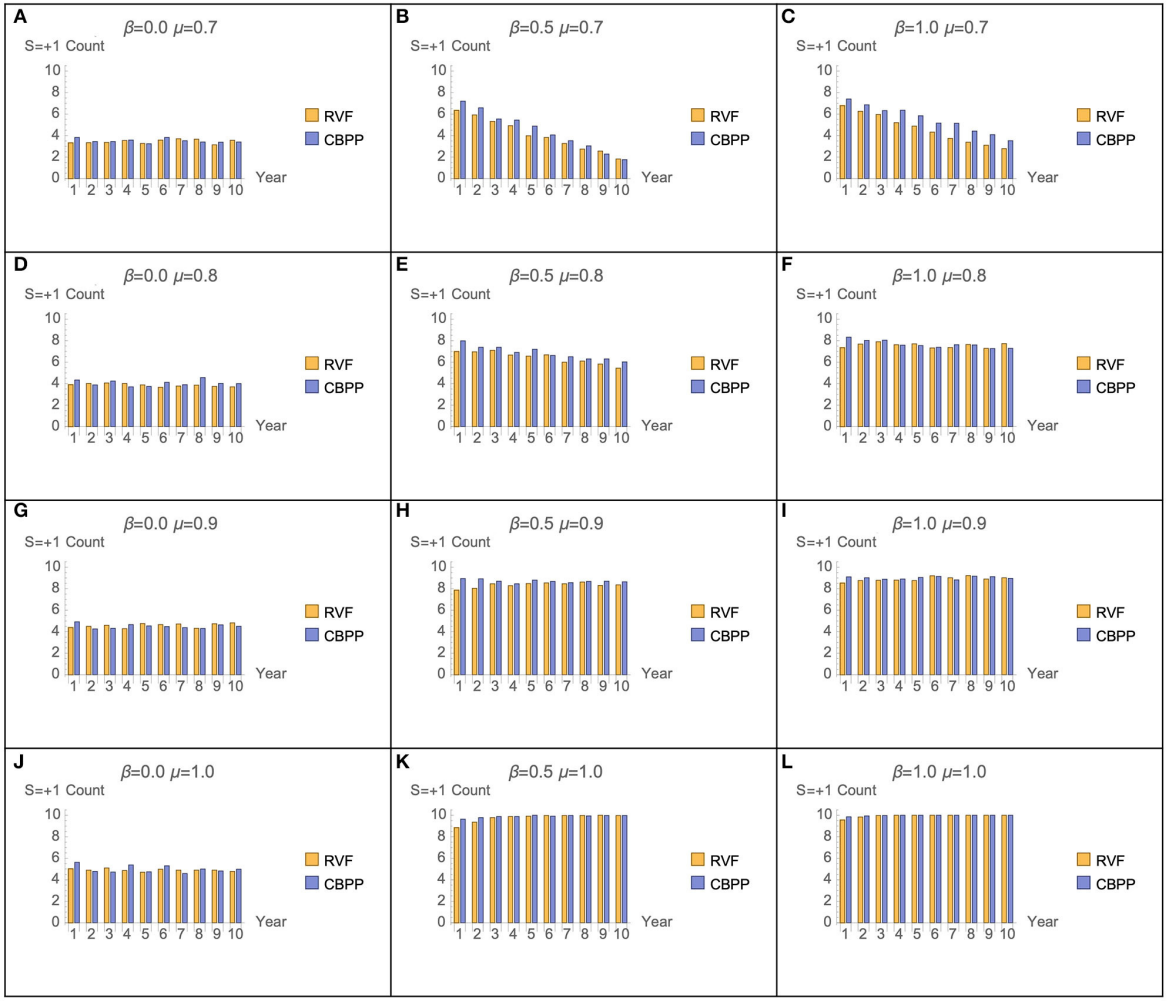
Ratio of RVF and CBPP vaccination decisions for β and μ parameter combinations (50 seeds used per parameter combination).

Figure 7 presents the number of RVF and CBPP vaccination doses given across all cattle owned by the heads-of-households assume various combinations of μ and β parameter values. Two distinct patterns are observed. First, across low levels of μ (memory) the number of CBPP vaccinations outnumber those for RVF for individual heads-of-households. This hints at a potential difference in the role of memory for vaccination decisions related to RVF (a cyclical, more predictable disease requiring once-a-lifetime vaccine) vs. CBPP (a less predictable disease requiring booster vaccines). In Figures 7B,C (where β = 0.5 and μ = 0.7, and β = 1.0 and μ = 0.7) the number of vaccination decisions for both RVF and CBPP declines over time. Second, the sum of the number of RVF and CBPP vaccinations is similar across the majority of parameter combinations. At low levels of rationality (β), irrespective of the level of memory, the proportion of vaccination decisions is <50%. Only once β is ≥0.5 and μ is ≥0.8 does this proportion pass 50%. We discuss these two results in more detail below.

## Discussion

The sensitivity analysis of the μ and β parameters enable a comparison across decision maker typologies. The graphs Figures 6C,J,H in represent decision makers with different combination of μ (memory) and β (rationality). Across the range of μ and β values graphs Figures 6C,J have parameter combinations at opposite extremes. Graph Figure 6H represents moderate levels of memory (μ = 0.9) and rationality (β = 0.5). This parameter combination may be not dissimilar to a person with “standard” levels of memory and rationality—strong but not perfect. At these strong, but not perfect, parameter values the number of cattle in the model is one of the most stable, after 350 weeks. The corresponding plot in Figure 7H (graph) has positive vaccine decisions for both CBPP and RVF at 80 percent or greater. The scenario with decision-makers with perfect memory of the past decision (μ = 1), but low rationality (β = 0) the graph Figure 6J in has the number of cattle in the model rapidly declining after 350 weeks. The corresponding graph in Figure 7J (graph) has positive vaccine choices at ~50 percent for both CBPP and RVF. For the opposite parameter mix of perfect rationality (β = 1) and relatively weak memory (μ = 0.7), graph c in Figure 6, the decline in the number of cattle alive in the model appears to decrease at an equally rapid rate after 350 weeks. Under this parameter scenario, graph of Figure 7F, the number of vaccination decisions start at 60% and then progressively decline to ~40%.

The lower efficacy of the CBPP vaccine, relative to the once-for-life RVF vaccine, and the need to decide annually whether to vaccinate cattle against CBPP increases the effect of changes in the μ and β parameters. Increasing β (rationality) values from 0.0 to 0.5 generated a 57 percent decrease in CBPP related cattle deaths, compared to a 6 percent decrease for RVF (Table 2). Increasing μ (memory) from 0.7 to 0.9 had a disproportionate positive effect on CBPP vaccine up-take compared to RVF (Figure 7). The relative lower effect of μ and β on RVF vaccine up-take is intuitive. While the risk of RVF is periodic (strongly associated with high rainfall and mosquito vectors), the life-time immunity given by the vaccine makes the need for heads-of-households to use “past experience” or memory of the most recent decision less important. Although the spread of both diseases are uncertain, RVF risk is periodically more certain following the onset of heavy rains generated by El Nino/Southern Oscillation weather pattern (30). If one believes that cattle will be exposed to high risk of RVF during the animal's life, then vaccinating early in the animal's life (whether or not the present risk of RVF is great) is sensible. The same cannot be applied to the annual booster for CBPP. The differences in uncertainty of disease risk for RVF and CBPP, as an explanation for the differing effects of μ and β, is further strengthened in light of the fact that no outbreaks of RVF or CBPP have recently been recorded in Samburu.

The *PastoralScape* ABM provides a realistic simulation of the environmental conditions of south-western Samburu by integrating historical measurements of the environment to drive mathematical models. This modeling realism of the natural environment provides a foundation to model livestock nutritional health, and herd mixing though common grazing of cattle within villages and herd movements. While the ABM presented captures “high-level” environmental change, it does so in a manner that motivates secondary dynamics of cattle health. Declines in available forage in and around villages prompts herdsmen to move cattle to protect against further livestock health declines. While model tuning and extended design is required to better capture the interactions between livestock non-disease health, herd movement and herd management, the current *PastoralScape* model provides a sound basis to identify the utility of using a RFIM to represent individual decision-making dynamics.

The *PastoralScape* model offers a platform to better understanding the relationship between natural systems and human decision making. Disease transmission is one such natural system. The results of the preliminary *PastoralScape* ABM highlight the effect of two different cognitive measures on vaccines with contrasting booster requirements. The effect of altering only β (rationality) or both μ (memory) and β (rationality) on the susceptibility of cattle to RVF and CBPP is meaningful. Modeling the effect of dynamic cognitive capacity, whether uniform or non-uniform across a population, on a range of decision contexts is supported by detailed experimental and non-experimental findings [(Choi and Iles, under review); (36)]. The incorporation of an RFIM for decision making within an ABM, as demonstrated by the *PastoralScape* model, provides a clear avenue to extend livestock disease modeling (6). Extending the *PastoralScape* model to include household income will allow for simulations of the effect of droughts on pastoralists' decision-making, including preventative livestock health measures.

The use of the RFIM, as specified by Bouchaud (37), in the current preliminary *PastoralScape* ABM provides a viable response to the need to more realistically model the temporal dynamics of binary decision making. By considering the short-run dynamics of changes in memory and rationality, dynamic decision making may be incorporated into ABMs. While such short-run changes are not currently implemented, the authors plan to do so in future work. The constructs of working memory capacity and fluid intelligence are measures that relate to β (fluid intelligence) and μ (working memory capacity). Working memory capacity measures the ability to recall salient information in the face of distractions (32, 50). Fluid intelligence measures one's ability to apply abstract reasoning (51). Dynamic changes in cognitive capacity due to stress is in keeping with the Mullainathan and Shafir's “scarcity thesis” (9).

Analyzing only μ and β (as two out of five RFIM parameters) for their effect on the probability of vaccine up-take and cattle mortality is deemed most manageable for such a preliminary model. In addition, the assumption of homogeneity of parameter levels aids the communication of the preliminary *PastoralScape* model. Consideration of the effects of the other three parameters (willingness to act, public information, and social network pressure) on livestock vaccine decision making is planned. The rapidly increasing combination of parameter combinations makes this difficult (26 combinations of five continuous parameters). The parameter μ (memory) and β (rationality) were selected first due to their relevance to the literature concerning individuals' internal barriers to experiencing poverty alleviation. The scarcity and aspiration failure these are two prominent examples (10, 52). Analysis of the effects of fluid intelligence (proxy for rationality) and working memory capacity (proxy for memory) among the Samburu shows that households in the lowest income quartile (ultra-poor households) have distinct effect on the likelihood of tick treatment and CBPP vaccine choice (Choi and Iles, under review).

The present research describes preliminary work in developing a fully coupled natural and human simulation that models livestock vaccine choice, herd management, and resulting causes of death. In addition, the model presented here provides a feasible alternate to the more common but limited assumption of a fully rational agent.

## Data Availability Statement

The raw data supporting the conclusions of this article will be made available by the authors, without undue reservation.

## Author Contributions

RI devised the project and the main conceptual ideas. MS worked out the technical details and wrote the code. OA provided the GIS data, while EL and CM verified the epidemiological and animal health submodels. RI drafted the manuscript with assistance from MS. OA, EL, and CM provided comments on the final draft. All authors contributed to the article and approved the submitted version.

## Conflict of Interest

The authors declare that the research was conducted in the absence of any commercial or financial relationships that could be construed as a potential conflict of interest.
